# FDoSR-Net: Frequency-Domain Informed Auto-Encoder Network for Arbitrary-Scale 3D Whole-Heart MRI Super-Resolution

**DOI:** 10.3390/bioengineering12020129

**Published:** 2025-01-30

**Authors:** Corbin Maciel, Qing Zou

**Affiliations:** 1 Department of Biomedical Engineering, The University of Texas Southwestern Medical Center, Dallas, TX 75390, USA; corbin.maciel@utsouthwestern.edu; 2 Division of Pediatric Cardiology, Department of Pediatrics, The University of Texas Southwestern Medical Center, Dallas, TX 75390, USA; 3 Advanced Imaging Research Center, The University of Texas Southwestern Medical Center, Dallas, TX 75390, USA; 4 Department of Radiology, The University of Texas Southwestern Medical Center, Dallas, TX 75390, USA

**Keywords:** 3D whole-heart MRI, super-resolution, deep learning, frequency-domain regularization

## Abstract

This work aims to develop a three-dimensional (3D) super-resolution (SR) network that would perform arbitrary-scale 3D whole-heart (WH) magnetic resonance imaging (MRI) super-resolution, while maintaining fine image details. One-hundred-twenty 3D WH MR volumes, acquired using four different sequences, are used in this study for training, validation, and testing. The proposed method utilizes a frequency-domain regularization in training to maintain fine image detail along with a 3D autoencoder framework. It is also trained in manner to enable it to perform arbitrary factor SR. The proposed method is compared against multiple super-resolution algorithms including two state-of-the-art deep learning methods referred to here as ACNS and TFC as well as nearest neighbor interpolation. The proposed method was evaluated quantitatively and compared against the competing methods with the mean result of the proposed method and the improvements provided by the proposed method (reported by mean percentage between the proposed method and all other competing methods) were recorded. The metrics of interest used for the quantitative comparison are peak signal-to-noise ratio (PSNR, mean = 34.10, mean percentage of improvement = 4.5%), structural similarity index measure (SSIM, mean = 0.94, mean percentage of improvement = 2.2%), mean squared error (MSE, mean = 0.00094, mean percentage of improvement = 48.2%), and root mean squared error (RMSE, mean = 0.024, mean percentage of improvement = 31.0%). Moreover, qualitative comparison was performed using multiple visual comparisons. The quantitative results achieved demonstrate that the proposed method regularly outperforms all other comparison methods. The visual comparisons demonstrate that the proposed method outperforms current state-of-the-art methods in preserving fine image details, as well as its ability to do so for multiple SR factors.

## 1. Introduction

Three-dimensional (3D) whole-heart (WH) magnetic resonance imaging (MRI) is a technique that allows observation and examination of the structure of the heart for patients with congenital and acquired heart diseases [1,2,3]. Many benefits are offered by this technique. One such benefit is that it is radiation-free [1,4] compared to computed tomography angiography (CTA). Secondly, the isotropic resolution of 3D WH MRI enables better visualization of the heart anatomy using multi-planar reconstruction (MPR). This includes the evaluation of small structures such as coronary arteries. Due to the requirements for the evaluation of small structures, high spatial resolution is necessary. However, the 3D technique presents one main limitation, which is the long acquisition time due to electrocardiogram (ECG) triggering and respiratory triggering [1,5]. This long acquisition time increases the probability of motion artifacts from respiratory motion occurring within the image and the discomfort for patients.

Several efforts have been made to decrease the scan time of 3D WH MRI, such as the work done in [6] where a dual phase technique was developed which allowed 3D WH images at both the systolic and diastolic phases to be acquired in the same amount of time as a single-phase sequence [6]. In [7] image-navigation was applied to the dual-phase technique proposed in [6] and lead to a further decrease in scan time. Methods such as these are from the sequence development perspective. Engineering approaches have also been developed to solve this problem. One such workaround that has the potential to decrease scan time is super-resolution (SR). SR uses the information from a low-resolution image to create a high-resolution image. Thus, because SR only requires a low-resolution scan to be acquired, scan time can be decreased. There are multiple ways to perform SR in MRI. One that is commonly implemented on MR scanners is zero-filling [8,9]. Unfortunately, this method is not particularly robust and is limited in its capacity to super-resolve an image [8]. Other interpolation methods such as linear interpolation can also be used to perform SR. However, these are still limited in their ability to perform SR, often leading to artifacts such as blurring and aliasing [10]. The limited application of these approaches and others related to them has led to the need for more robust SR methods to be developed.

**Figure 1:**
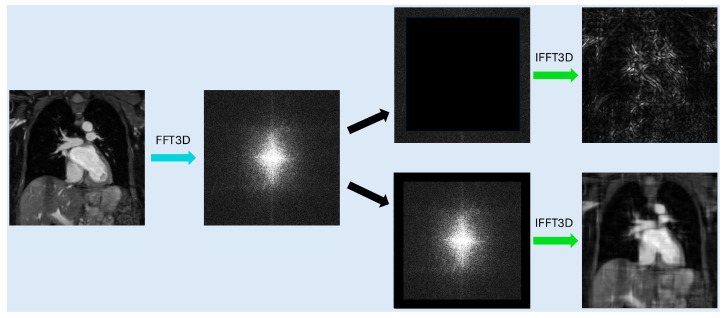
Illustration of the synthetic frequency-domain, with FFT3D indicating a 3D Fast Fourier Transform and IFFT3D indicating an 3D inverse Fast Fourier Transform. One 2D slice in the 3D volume is selected to showcase the synthetic frequency-domain. The synthetic frequency-domain is obtained by performing a 3D Fourier Transform on the given image. Removing the outer portion of the synthetic frequency-domain eliminates much of the fine detail, while removing the center leaves only the fine image detail. The black arrows indicate separating the frequency-domain into its high and low-frequency components.

As the field of artificial intelligence has advanced in recent years, deep learning methods have emerged as a robust tool to perform complex computer vision tasks, with many being developed to perform SR for both natural images and medical images [11,12,13,14,15]. Deep learning methods have made great strides in overcoming the limitations of the basic interpolation methods. However, very limited research has been done for the application of 3D WH MRI [16,17,18]. Furthermore, cardiac MRI presents a unique challenge in that the heart contains anatomy that is both small and complex. The method proposed in this study, the frequency-domain informed SR network (FDoSR-Net), uses the information from the frequency-domain to inform the network during training. It is important to note that within this study it is assumed that only the reconstructed real-valued MR image is accessible, as is usually the case in clinical practice. Thus, the complex-valued frequency-domain cannot be used here. However, it is known that the frequency-domain of the MR image contains valuable information, especially the outer part of the raw k-space data that captures the structural details of the images. Therefore, to enjoy the benefits of the information provided in the frequency-domain, we propose to take the Fourier Transform of the reconstructed real-valued MR images to get the synthetic frequency-domain. We use the word “synthetic” here to distinguish it from the raw k-space. Obtaining the synthetic frequency-domain in this way inverts the information contained therein, such that the center of the synthetic frequency-domain contains information regarding the image detail (Figure 1), and the outer portion of the synthetic frequency-domain contains information relating to the image contrast.

Currently, most SR deep learning methods are limited in their practical applicability because they can only be trained to perform SR for one factor at a time [18,19,20,21]. Recently, there have been a few approaches proposed for arbitrary factor SR [22,23,24,25]. Motivated by arbitrary factor SR, the FDoSR-Net learns to perform arbitrary factor SR by being trained on MR volumes that are randomly undersampled by SR factors between 2 and 4, including non-integer factors.

A few SR deep learning methods have been proposed for 3D WH MRI. In [16] an approach using a 3D U-Net is proposed and adapted to perform SR for 3D WH MRI. While it demonstrates promising results, very little was done to optimize the 3D U-Net for 3D WH MRI. Thus, the work done in [16] leaves much to be done in bridging the gap toward clinical applicability. In [17] an unsupervised skip connection network is proposed for SR of 3D WH MR volumes. This method seeks to solve the issue of limited 3D WH MRI training datasets that often exists. This method demonstrates strong performance of the SR task, but the need to be retrained for any given MR volume limits its practicality. The work done in [18] is a comparative study of popular SR deep learning methods extended to 3D WH MRI. The proposed method seeks to address the limitations of previous 3D WH MRI deep learning SR approaches through the implementation of the following novelties. First, arbitrary scale SR is implemented within the proposed framework, thereby enhancing its practicality in a clinical setting by eliminating the need to be trained multiple times. Second, the proposed method utilizes a frequency-domain regularization in the loss function. This frequency-domain regularization enables the proposed method to better preserve fine image details.

In this study the proposed FDoSR-Net implements a frequency-domain regularization for the maintenance of fine image detail, and it is trained to perform arbitrary factor SR. The proposed method utilizes an autoencoder framework [26] for super-resolution due to its robust performance for many complex computer vision tasks [27,28,29,30]. The proposed method achieves high-quality image output and maintains small anatomical structures in the heart well. This is demonstrated in this work through image quality metrics such as peak signal-to-noise ratio (PSNR), structural similarity index measure (SSIM), mean squared error (MSE), and root mean squared error (RMSE). The results of which are subsequently compared against other state-of-the-art deep-learning-based super-resolution methods [19,26]. Visual comparisons are also provided to further illustrate the efficacy of the FDoSR-Net.

## 2. Materials and Methods

### 2.1. Datasets

In this study, 120 MR volumes acquired from patients or volunteers were used in the training and testing of the FDoSR-Net. The patient MR volumes were acquired clinically for diagnostic purposes and the volunteer MR volumes were collected under the approval of the local Institute Review Board. All the data was acquired on a dedicated 1.5 T cardiac MR scanner (Philips, Best, Netherlands). In our institute, four different sequences are used in routine clinical practice for 3D WH MRI. The MR volumes are evenly distributed among the four different MR sequences; thus, 30 MR volumes exist for each acquisition type. Two of the four sequences were applied in cases where contrast agents were not used. One was an ECG and respiratory triggered relaxation-enhanced angiography without contrast and triggering (REACT) [31] sequence. The REACT sequence is a 3D magnetization-prepared non-balanced dual-echo Dixon technique, coupled with cardiac triggering and respiratory navigation. The in-depth sequence description can be found in [31]. The other is a fat-saturated ECG and respiratory triggered 3D balanced steady-state-free-procession (non-contrast enhanced SSFP or NCSSFP) sequence. Two additional sequences were used to obtain scans with contrast enhancement. The first is an ECG and respiratory triggered 3D balanced steady-state-free-procession (SSFP) sequence that was used with Gadolinium contrast. The second is an inversion recovery (IR) prepared ECG and respiratory triggered 3D balanced steady-state-free-procession (IRSSFP) sequence that was used with Ferumoxytol.

Each subgroup of 30 MR volumes was divided into groups of 20, 5, and 5 for training, validation, and testing. Therefore, the total number of MR volumes was 80, 20, and 20 for training, validation, and testing respectively. Prior to training, the training and validation MR volumes were randomly downsampled by random factors between 2 and 4. This enabled the FDoSR-Net to learn arbitrary scaling factor SR. To thoroughly test the performance of the FDoSR-Net, the testing MR volumes were downsampled by different factors between 2 and 4 in this study.

### 2.2. Proposed Super-Resolution Framework

**Figure 2:**
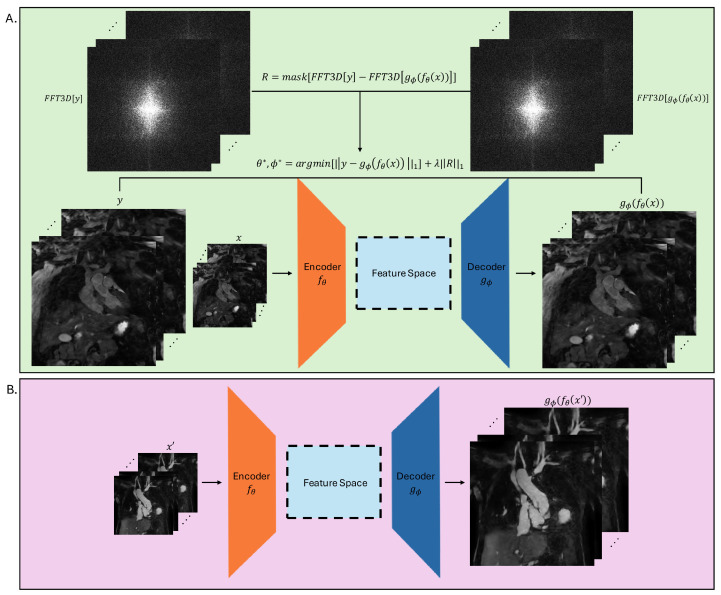
An illustration of the workflow of the FDoSR-Net. For the training step shown in (**A**), the low-resolution MR volume is input to the encoder. The encoder then produces feature vectors which are then used by the decoder to produce a high-resolution MR volume. The frequency-domain penalization term is then calculated using the FFT3D and a mask function. The loss function is then calculated, and the network parameters are updated. (**B**) illustrates the testing step, where a low-resolution image is super-resolved by the trained network.

The FDoSR-Net consists of a 3D encoder $f_{\theta }$, and a 3D decoder $g_{\phi }$, with θ and ϕ as learnable network parameters of the encoder and decoder respectively. The details of the encoder and the decoder are given in the next section. In the training stage, a low-resolution 3D MR image *x* is provided as input to $f_{\theta }$. Feature vectors from *x* are then extracted by $f_{\theta }$, and the feature vectors $f_{\theta }\left(x\right)$, are input into $g_{\phi }$, which then outputs the high-resolution image, $g_{\phi }\left(f_{\theta }\left(x\right)\right)$. The following minimization problem is then solved to update the network parameters:

(1) $$\theta^{*},\phi^{*}=argmin_{\theta ,\phi }[||y-g_{\phi }\left(f_{\theta }\left(x\right)\right){\left|\right|}_{1}{]+\lambda \cdot ||R||}_{1}$$

where *y* is the high-resolution ground truth image, *R* is the frequency-domain penalization term, and λ is the regularization hyperparameter that weights the balance between the data-consistency term and the frequency-domain penalization term. The L1 loss, shown in Equation (1) as ${\left||...|\right|}_{1}$, is used for both the data consistency and and regularization term. This is because the L1 loss does not heavily penalize large differences between voxels, allowing it to better preserve fine details of an image. Furthermore, the implementation of the L1 loss is simple and efficient. The frequency-domain penalization term *R* is defined as follows:

(2) $$R=mask[FFT3D\left[y\right]-FFT3D\left[g_{\phi }\left(f_{\theta }\left(x\right)\right)\right]]$$

where FFT3D indicates the 3D Fast Fourier Transform and mask represents the binary mask function used to select the center frequency-domain information. Respecting the mask, it is applied to zero-out the central portion of the frequency domain, while the data consistency term is in the imaging domain and no mask is applied to it. Furthermore, the mask size was determined heuristically, to minimize contrast information and maximize tissue border information. In this work, 30% of the outer k-space was chosen to be zeroed out. An illustration of this proposed framework can be seen in Figure 2.

The whole super-resolution framework was implemented using the PyTorch library, this includes an FFT or IFFT calculation. All the experiments were done on a workstation equipped with an NVIDIA RTX A6000 48 GB GPU. The training was carried out over 2400 epochs, with an initial learning rate of $10^{-4}$ and was updated every 200 epochs. Both networks were initialized randomly. Optimization of the parameters was based on Adam optimization [32]. The testing phase of the network requires an average of less than half minute per image volume. The cost to train the FDoSR-Net is 15.67 GB and the cost of inference is 15 GB. The training time for the network is 22 h.

### 2.3. Network Details

**Figure 3:**
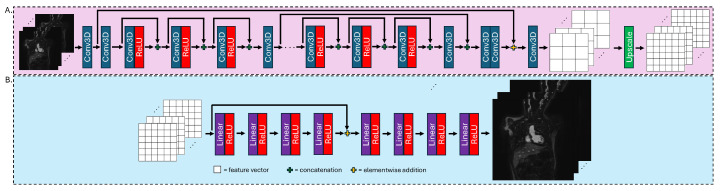
A schematic of the encoder (**A**) and decoder (**B**). The encoder uses series of residual dense blocks (RDB) to extract feature vectors from the low resolution images. Each RDB consists of three convolutions and three ReLU acitvation functions, followed by one additional convolution. The initial convolutions are used to extract global features and the final convolution is used to fuse global and local features, thus yielding the final feature vectors. The decoder utilizes a series of linear operators together with ReLU activation functions to produce the final high-resolution image.

The encoder $f_{\theta }$, is a residual dense network, with low-resolution images provided as input. The first step in $f_{\theta }$ is shallow feature extraction, performed using two 3D convolutions. This initial step preserves information relating to the spatial structure of the image. The output from the shallow feature extraction is input into a series of 8 residual dense blocks (RDB). Each RDB is comprised of 3 layers used to extract local features. Each layer is made up of a 3D convolution followed by a ReLU activation function [33]. The input and output of each layer are concatenated and input into the next layer. After the third layer of each block, another 3D convolution is performed to fuse the local features extracted by the given block. The output from each RDB is then concatenated together and passed through two 3D convolutions. The output of this step is then combined with the output of the first shallow feature convolution using elementwise addition. This enables the network to learn global features. Finally, to produce $f_{\theta }\left(x\right)$, a final 3D convolution layer with feature dimension 128 and an upscale layer are performed, providing a feature vector corresponding to each voxel in the super-resolved image with each vector containing 128 elements. Of important note is that the encoder does not utilize any pooling (i.e., downsampling) operators or any normalization operators. This is done to eliminate information loss due to pooling and to enhance computational efficiency as batch normalization costs as much as a convolution [34]. Moreover, there are no skip connections between the encoder and decoder. A detailed schematic of the encoder can be seen in Figure 3A and an outline of the encoder architecture can be found in Appendix A Table A1.

The decoder $g_{\phi }$, is a multi-layer perceptron (MLP) comprised of 8 total layers. Each layer is realized by a linear operator together with a nonlinear ReLU activation function, yielding the final high-resolution image, $g_{\phi }\left(f_{\theta }\left(x\right)\right)$. The selection of an MLP as the decoder network is twofold. First, MLPs have demonstrated the ability to maintain spatial resolution for tasks such as SR and image restoration [35,36]. Second, the use an MLP permits the FDoSR-Net to perform arbitrary factor SR. By training the MLP using data undersampled by multiple scaling factors, the MLP is able to learn a scaling function rather than discrete transforms for a single scaling factor. Moreover, the resolution of the resulting image is based on the number of elements in the feature vectors, which remains a constant in the FDoSR-Net for each scaling factor. Figure 3B provides a schematic of the architecture of the decoder. Table A2 outlines the architecture of the decoder and can be found in Appendix A.

### 2.4. Quantitative and Visual Analysis

In this study the quantitative metrics used to assess the performance of the FDoSR-Net were PSNR, SSIM, MSE, and RMSE. Each of these metrics analyze the closeness of a given image to its ground truth. PSNR is interpreted as follows, the larger the PSNR, the closer a given image is to the ground truth. SSIM produces a value between 0 and 1, where 0 indicates no similarity between the two images and 1 indicates that the images are the same. MSE and RMSE also measure the similarity between the ground truth image and a given image. The larger the MSE and RMSE the more different the images are. The smaller the MSE and RMSE the closer the two images are in resemblance, with a value of 0 indicating a perfect match. These metrics serve as a measure of image quality between the output of the methods used herein and the ground truth.

A thorough visual analysis was also performed to assess the performance of the FDoSR-Net qualitatively. The output of the FDoSR-Net was compared against the high-resolution and low-resolution ground truth images, to assess the overall performance of the proposed method. Additionally, to evaluate the output of the FDoSR-Net against other state-of-the-art deep-learning-based super-resolution methods [19,26], a visual comparison was created comparing the output of each method against the high-resolution ground truth. Visual comparisons highlighting the effectiveness of the frequency-domain regularization, and the robustness of the FDoSR-Net were also included.

## 3. Results

### 3.1. Effectiveness of the Frequency-Domain Regularization

**Figure 4:**
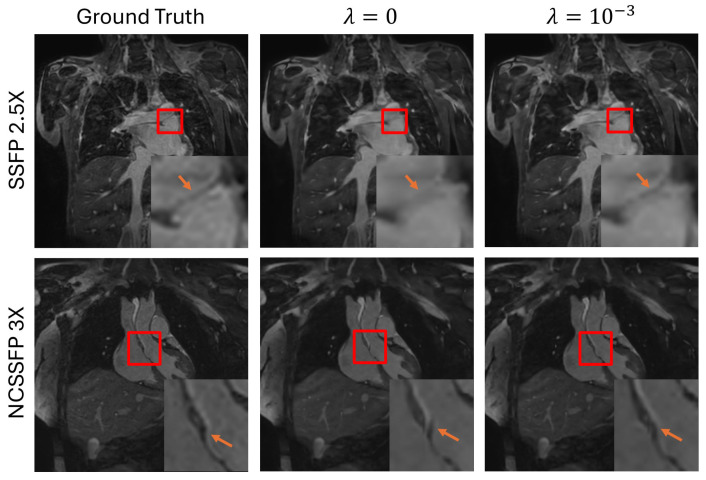

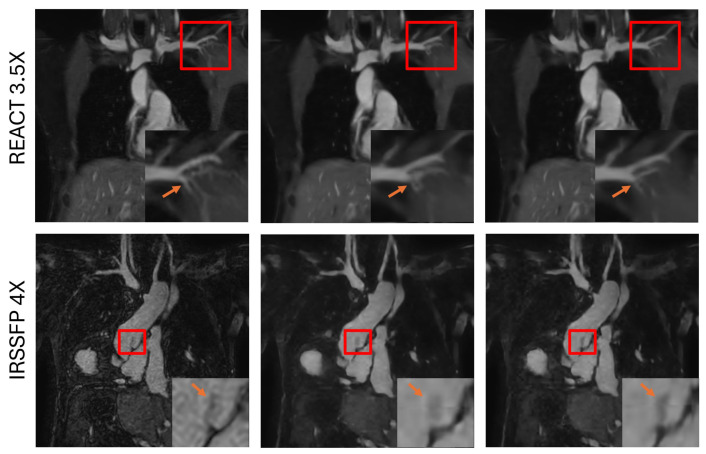
A visual comparison demonstrating the effect of the frequency-domain penalization. The red squares designate the region of interest that indicates clear difference, and the orange arrows indicate the anatomy of interest within the regions that show clear differences.

In this section, the effectiveness of the frequency-domain regularization is studied. The results can be found in Figure 4. Images from different sequences at different SR factors are shown. Each row in the figure shows a different subject from one of the 4 sequences used and a zoomed-in image showing the differences of fine detail of various aspects of the anatomy is also displayed in the right-lower corner of each image. In the first row, an exemplary image acquired using the SSFP sequence was super-resolved with a factor of 2.5. In this case, the border of two different pieces of anatomy is still clear when frequency-domain regularization is used to inform training, whereas when the frequency-domain regularization is ignored, the border is blurred out. The next row shows a portion of the aorta where without frequency-domain regularization, there are artifacts which do not exist in the ground truth image. However, the aorta is completely resolved in the image with frequency-domain regularization, as is the case in the ground truth. The final two rows show vessels in the shoulder and a portion of the ascending aorta, respectively. In both cases, the anatomy is either inaccurate or difficult to visualize when frequency-domain regularization is not used to inform training. However, when frequency-domain regularization is applied, it provides a more accurate and clear depiction of the respective anatomy. Furthermore, Table 1 demonstrates the when the frequency-domain is included in training the network provides better performance.

### 3.2. Determination of the Regularization Hyperparameter

To determine the optimum regularization hyperparameter λ, we performed an ablation study by testing different values of the regularization hyperparameter. Specifically, we trained the network with $\lambda =10^{-2},10^{-3},10^{-5},10^{-10},$ and $10^{-15}$. For $\lambda =10^{-2}$ the training diverged and hence $10^{-2}$ did not work. However, each of the other regularization hyperparameters led to convergence. The trained models with different regularization hyperparameters were applied on the testing data for super-resolution. The PSNR and SSIM were calculated and compared for each case, and the results are shown in Table 2. From the table, we see that in most cases, $\lambda =10^{-3}$ achieved the best PSNR and SSIM. Based on these results, $\lambda =10^{-3}$ was used in this work.

It is important to note other hyperparameters of concern within this study such as the learning rate and the number of training epochs. The learning rate was chosen such that it minimized time to convergence. If the learning rate was too small, the network required long training times for convergence. With that, if the learning rate was too large, the network diverged. The number of epochs was chosen such that training time was minimized while maintaining strong model performance. Moreover, other characteristics of the network such as the number of layers were optimized to the best balance between GPU memory usage and network performance.

### 3.3. Illustration of the Proposed Framework

**Figure 5:**
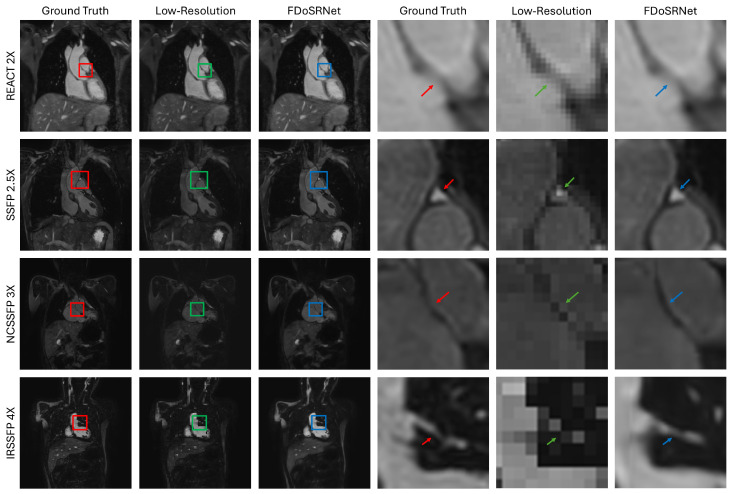
Illustration of the proposed method. Various anatomical details of the high-resolution ground truth, low-resolution images, and output of the FDoSR-Net are compared from each sequence at multiple SR factors. The red box and arrow correspond to the ground truth, the green box and arrow correspond to the low-resolution image, and the blue box and arrow correspond to the output of the FDoSR-Net.

To provide a clear illustration of the efficacy of the FDoSR-Net, a visual comparison was performed between the high-resolution ground truth, the low-resolution input images, and the high-resolution output of the proposed method. Figure 5 shows images from 4 different subjects at 4 different SR factors, from the 4 different MR sequences used. The zoomed-in images are used to highlight how the proposed framework super-resolved the low-resolution input and preserved the fine features. From the figure, it is demonstrated that the small anatomical details become visible when the FDoSR-Net performs SR on the low-resolution image. Moreover, it shows close resemblance between the output from the FDoSR-Net and the high-resolution ground truth. Examining the top row of Figure 5, the boundaries of the ascending aorta are very pixilated and difficult to distinguish in the low-resolution image, whereas those in the output of the FDoSR-Net are well defined and close to the ground truth. Other examples shown in the figure also illustrate the proposed framework’s ability to resolve the fine details of the heart, including the coronary artery.

### 3.4. Evaluation of the Robustness of the Proposed Framework

**Figure 6:**
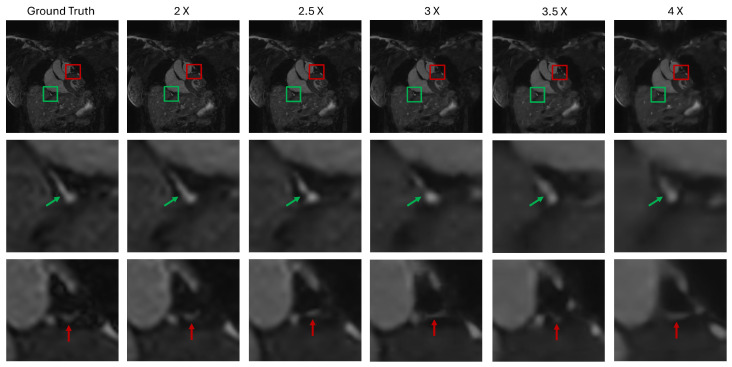
Showcase of the robustness of the FDoSR-Net. A slice of an NCSSFP image is shown, highlighting the preservation of small anatomical detail across multiple SR factors. The green box and arrow highlight tissue between the liver and right atrium and the red box and arrow show show a piece tissue adjacent to the aorta.

In the super-resolution task, as the SR factor increases the image quality of SR methods can significantly decrease because less prior information is available. In this section, we show that the proposed method maintains reasonable image quality, even at large SR factors. This is demonstrated in Figure 6 where an image slice acquired using the NCSSFP sequence is shown for each SR factor. The region outlined in green shows a piece of tissue between the liver and the right atrium. The region outlined in red illustrates a portion of the heart adjacent to the aorta. Examining the case of SR factor 2, FDoSR-Net closely resembles the ground truth. When moving to the cases of SR factors 2.5, and 3, there is some noticeable difference between the FDoSR-Net output and ground truth. However, most of the image detail is preserved, thus maintaining good resemblance between the images. This trend continues with each of the remaining high SR factors. Even at 4X SR, good image quality is maintained and many fine image details are still preserved.

### 3.5. Cross-Validation

To demonstrate the generalizability of the train/validation/test split strategy that we used in this work, we also performed a 5-fold cross-validation strategy for comparison. To evaluate the effectiveness of the resulting images, the metrics PSNR, SSIM, MSE, and RMSE were used. Examining the results found in Table 3, it can be seen that the PSNR, SSIM, MSE, and RMSE from the 5-fold cross-validation match closely with those achieved by the train/validation/test split strategy in Table 4. Furthermore, as the standard deviations indicate there is minimal variability across the metrics for each group at each SR factor. Thus, the proposed method demonstrates consistent performance for each data split in this experiment.

### 3.6. Comparison Against State-of-the-Art Methods

In this experiment, the proposed method was compared quantitatively and qualitatively against nearest neighbor interpolation and two state-of-the-art deep learning methods one being the autoencoder-inspired convolutional network-based SR method (ACNS) [26] and the other being the method proposed in [19] referred to here as the tensor feature clustering method (TFC). The ACNS is comprised of an encoder and decoder. The encoder takes in the low-resolution images and extracts features from the low-resolution images. The decoder then outputs the high-resolution images from the feature maps. The encoder of the ACNS does not use pooling layers, thus decreasing information loss due to parameter reduction. Furthermore, because the ACNS implements an autoencoder framework, there are fewer nodes used in the hidden layers. This allows the ACNS to maintain low computation times. The TFC uses tensors created from image patches of the low-resolution MR volume to produce filters and labels. The labels consist of clustered features extracted from the low-resolution image. The filters approximate the ground truth high-resolution intensity from the associated low-resolution patch. Thus, the filters are applied to the low-resolution patch for each label, yielding the high-resolution output. This enables the TFC to perform high quality SR and maintain tissue boundaries. The TFC also implements a patch span reduction method [19], allowing it to be trained on small datasets without model degradation.

The quantitative comparison was performed using PSNR, SSIM, MSE, and RMSE. The results of this comparison can be seen in Table 4. From the table, it is shown that in most cases, FDoSR-Net is able to achieve results comparable to or better than all other competing methods.

**Figure 7:**
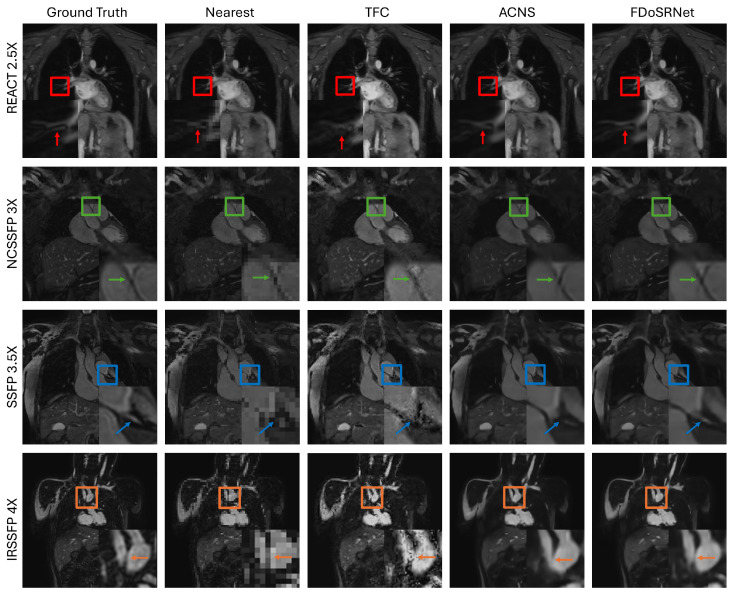
A visual comparison of the proposed method against nearest neighbor interpolation and two state-of-the-art deep learning methods, ACNS [26] and TFC [19]. Slices from each sequence and multiple SR factors are shown, demonstrating the ability of the FDoSR-Net to maintain small anatomical detail compared to other methods. Each colored box and arrow corresponds to the given sequence and SR factor; red = REACT 2.5X, green = NCSSFP 3X, blue = SSFP 3.5X, and orange = IRSSFP 4X.

A visual comparison shown in Figure 7 provides a qualitative analysis of the proposed method for 4 SR factors with images obtained from 4 different sequences. The top row of Figure 7 shows images acquired using the REACT sequence and super-resolved by a factor of 2.5. The highlighted portion shows distinct vessels that are close to the ground truth. However, each comparison method merges the vessels together. Pulmonary vessels such as these are examined when evaluating patients for certain heart conditions. Thus, inaccurate depictions of the vessels in Figure 7 could affect the physician’s ability to provide a correct diagnosis. The remaining examples in Figure 7 also show that the FDoSR-Net preserves fine details, whereas these details are either blurred, noisy, or inaccurate in the other methods, when compared to the ground truth images.

From the comparison study, both the quantitative and qualitative results showed that the performance of FDoSR-Net is comparable to other state-of-the-art methods and that it better preserves small anatomical details that are often lost by other methods.

## 4. Discussion and Conclusions

In this study we proposed an auto-encoder-based deep learning model to perform arbitrary factor SR using a frequency-domain regularization for the maintenance of small anatomical details. The proposed method was shown to perform at a level comparable to other state-of-the-art deep learning SR methods. This is shown quantitatively by the results provided in Table 4. Furthermore, the proposed method demonstrated a strong ability to preserve fine details within the MR images. This was shown in the visual comparisons in Figure 4 and Figure 7 where the benefit of frequency-domain regularization is illustrated, and the FDoSR-Net is compared against other methods.

One of the main novelties of the FDoSR-Net is the utilization of a frequency-domain penalization during learning. To the best of our knowledge this is the first time such an approach has been implemented in an SR algorithm for 3D WH MRI. By adding this term to the loss function during training, the FDoSR-Net is able to maintain fine details within the heart anatomy.

Arbitrary factor SR is a technique that can greatly increase clinical relevance and practicality as there is no need to retrain networks for different SR factors. This technique has been applied in [22] for 3D brain MRI using implicit neural representation (INR). The ArSSR framework [22] considers arbitrary SR factors during the training and hence provides the ability for arbitrary factor SR during the testing stage. Another approach taken in [23] was to develop a module that can be plugged into existing SR networks to perform arbitrary factor SR. This module creates a factor customized feature kernel to adapt the extracted features to the desired factor [23]. Applying this module to various state-of-the-art deep learning methods enhances model performance at various SR factors. The FDoSR-Net achieves arbitrary factor SR by randomly undersampling MR volumes during the training. Thus, we can achieve arbitrary factor SR without increasing the complexity of the network and maintain strong model performance.

Our model also adapted the autoencoder framework in [26] to perform SR on 3D WH MR images. The simplicity of the autoencoder framework enabled a significant increase in computational speed. By extending this method to 3D WH MRI, the FDoSR-Net gained the benefits provided by the autoencoder framework.

The main limitation of the proposed framework is its high dependency on large, high-quality labeled MR volumes. Our organ of interest is the heart, which is one of the most challenging organs to image using MRI due to motion. Thus, acquiring high quality ground-truth data is challenging. In our work, the training MR volumes are acquired in an undersampled fashion and reconstructed using a vendor-provided compressed sensing method. This leads to degradation in the images, hence training MR volumes used in this work contain greater noise and undersampling artifacts in the images. The model also learns these patterns, leading to some concerns in applying the model when it is trained on imperfect data. In the future, we will further evaluate the proposed framework when fully sampled training data becomes available. An additional limitation of this work is the lack of considering information across the various types of contrast to enhance training. In future work, we would like to explore the implementation of multi-contrast information such as those used in [37,38] to further enhance the performance of the proposed method.

In conclusion, we developed a deep learning framework to perform SR on 3D WH MR images. To preserve the small anatomical details, a frequency-domain regularization was applied to inform training. The autoencoder framework was adapted to perform SR of 3D MR volumes and the model was trained in a manner to enable it to perform arbitrary factor SR. The results achieved by the FDoSR-Net indicate that it has the potential to be a clinically useful tool for decreasing acquisition time in 3D WH MRI while still producing high-resolution images.

## Figures and Tables

**Table 1:** Quantitative comparison of the FDoSR-Net with and without the frequency-domain regularization. The Bold values indicate the best PSNR and SSIM for the given SR factor.

λ=0	$\lambda =10^{-3}$	λ=0	$\lambda =10^{-3}$
PSNR		SSIM	
IRSSFP			
34.33	**34.86**	0.91	**0.92**
NCSSFP			
34.11	**34.61**	**0.94**	**0.94**
REACT			
34.00	**34.01**	**0.96**	**0.96**
SSFP			
33.34	**33.65**	**0.94**	**0.94**

**Table 2:** PSNR and SSIM for the various values tested for the regularization term. The Bold values indicate the best PSNR and SSIM for the given SR factor. Of important note is that $10^{-2}$ diverged during training.

λ	$10^{-2}$	$10^{-3}$	$10^{-5}$	$10^{-10}$	$10^{-15}$	$10^{-3}$	$10^{-5}$	$10^{-10}$	$10^{-15}$
Metric	PSNR					SSIM			
IRSSFP									
2X	Diverged	40.43	**40.45**	40.19	39.74	**0.9758**	0.9754	0.9749	0.9755
2.5X	Diverged	**35.74**	34.60	35.38	34.61	**0.9236**	0.9206	0.9220	0.9231
3X	Diverged	**34.81**	33.80	34.08	33.76	**0.9196**	0.9159	0.9159	0.9186
3.5X	Diverged	**32.65**	32.21	31.60	31.97	**0.8962**	0.8940	0.8924	0.8943
4X	Diverged	**30.66**	30.08	29.86	30.17	**0.8630**	0.8584	0.8614	0.8624
NCSSFP									
2X	Diverged	**40.31**	39.96	39.86	39.92	**0.9862**	0.9858	0.9858	0.9857
2.5X	Diverged	**34.20**	33.66	33.90	33.82	**0.9479**	0.9456	0.9472	0.9468
3X	Diverged	**34.62**	34.07	34.16	34.06	**0.9493**	0.9461	0.9466	0.9475
3.5X	Diverged	**33.09**	32.56	32.50	32.41	**0.9320**	0.9285	0.9290	0.9292
4X	Diverged	**30.83**	30.53	30.65	30.45	0.9017	0.8980	**0.9036**	0.9005
REACT									
2X	Diverged	**40.48**	40.39	39.64	40.20	**0.9918**	0.9915	0.9916	0.9917
2.5X	Diverged	**36.87**	36.02	35.95	36.26	**0.9810**	0.9793	0.9803	0.9799
3X	Diverged	**32.69**	32.37	32.30	32.46	0.9556	0.9530	**0.9558**	0.9555
3.5X	Diverged	**31.16**	30.78	30.80	30.74	**0.9417**	0.9383	0.9414	0.9403
4X	Diverged	**28.84**	28.68	28.44	28.70	**0.9144**	0.9114	0.9137	0.9138
SSFP									
2X	Diverged	39.53	**39.76**	39.19	39.31	**0.9871**	0.9868	0.9868	0.9867
2.5X	Diverged	**33.46**	32.68	33.09	33.16	**0.9475**	0.9442	0.9465	0.9462
3X	Diverged	**33.85**	33.24	33.58	33.24	**0.9492**	0.9465	0.9472	0.9479
3.5X	Diverged	**31.99**	**31.99**	31.24	31.86	**0.9313**	0.9296	0.9282	0.9299
4X	Diverged	29.42	29.44	29.15	**29.46**	0.8928	0.8890	0.8911	**0.8932**

**Table 3:** Resulting PSNR, SSIM, MSE, and RMSE of the 5-fold cross-validation for each scaling factor.

	PSNR	SSIM	MSE $\left(10^{-4}\right)$	RMSE $\left(10^{-2}\right)$
2X	40.18 ± 0.89	0.99 ± 0.00	1.8 ± 0.5	1.1 ± 0.1
2.5X	34.80 ± 0.91	0.95 ± 0.00	6.6 ± 0.9	2.1 ± 0.1
3X	33.67 ± 0.74	0.94 ± 0.01	7.9 ± 0.9	2.3 ± 0.1
3.5X	31.90 ± 0.84	0.92 ± 0.01	11.6 ± 1.1	2.8 ± 0.2
4X	29.95 ± 0.90	0.89 ± 0.01	19.1 ± 2.0	3.6 ± 0.2

**Table 4:** Quantitative comparison of the FDoSR-Net against other methods. The Bold values indicate the best PSNR, SSIM, MSE, and RMSE for the given SR factor.

Method	FDoSR_Net	ACNS	TFC	Nearest
Metric	PSNR			
2X	**40.19**	39.86	37.86	37.15
2.5X	**35.07**	34.32	33.43	32.55
3X	**33.99**	33.46	30.42	32.77
3.5X	**32.22**	32.02	27.95	30.98
4X	29.94	**30.06**	27.39	30.46
SSIM				
2X	**0.99**	**0.99**	0.98	0.96
2.5X	0.95	0.95	**0.96**	0.90
3X	**0.94**	**0.94**	0.92	0.90
3.5X	**0.93**	0.92	0.88	0.87
4X	**0.89**	**0.89**	0.86	0.84
MSE ($10^{-4}$)				
2X	**1.7**	2.1	45.4	4.8
2.5X	**6.0**	6.7	46.6	13.9
3X	**6.8**	7.4	42.4	13.3
3.5X	**9.9**	10.0	68.6	18.6
4X	17.4	**16.5**	50.0	37.3
RMSE ($10^{-2}$)				
2X	**1.1**	**1.1**	5.3	1.8
2.5X	**2.0**	2.1	5.7	3.1
3X	**2.2**	2.3	5.4	3.0
3.5X	**2.6**	**2.6**	6.1	3.6
4X	3.4	**3.3**	5.8	4.2

## Data Availability

The data presented in this study are available on request from the corresponding author.

## Appendix A. Architecture of the Encoder and Decoder

**Table A1:** Architecture of the the encoder used in the proposed method.

Layer	Input Size	Kernal Size	Padding	Stride	Output Size
1	1	3	1	1	64
2	64	3	1	1	64
The following is the same for each RDB.					
2, 5, 8	128	3	1	1	64
3, 6, 9	192	3	1	1	64
4, 7, 10	256	3	1	1	64
4, 7, 10	256	1	0	1	64
End of RDBs					
11	512	1	0	1	64
12	64	3	1	1	64
13	64	3	1	1	128

**Table A2:** Architecture of the the decoder used in the proposed method.

Layer	Input Size	Output Size
1	128	256
2	256	256
3	256	256
4	256	128
5	128	256
6	256	256
7	256	256
8	256	1
